# Screening mitochondria-related biomarkers in skin and plasma of atopic dermatitis patients by bioinformatics analysis and machine learning

**DOI:** 10.3389/fimmu.2024.1367602

**Published:** 2024-05-07

**Authors:** Huiwen Yu, Jiaying Lin, Jinping Yuan, Xianqi Sun, Chen Wang, Bingxue Bai

**Affiliations:** Department of Dermatology, The Second Affiliated Hospital of Harbin Medical University, Harbin, Heilongjiang, China

**Keywords:** atopic dermatitis, mitochondria, diagnostic biomarker, mitochondrial metabolism, immune infiltration, circulating cell-free mitochondrial DNA

## Abstract

**Background:**

There is a significant imbalance of mitochondrial activity and oxidative stress (OS) status in patients with atopic dermatitis (AD). This study aims to screen skin and peripheral mitochondria-related biomarkers, providing insights into the underlying mechanisms of mitochondrial dysfunction in AD.

**Methods:**

Public data were obtained from MitoCarta 3.0 and GEO database. We screened mitochondria-related differentially expressed genes (MitoDEGs) using R language and then performed GO and KEGG pathway analysis on MitoDEGs. PPI and machine learning algorithms were also used to select hub MitoDEGs. Meanwhile, the expression of hub MitoDEGs in clinical samples were verified. Using ROC curve analysis, the diagnostic performance of risk model constructed from these hub MitoDEGs was evaluated in the training and validation sets. Further computer-aided algorithm analyses included gene set enrichment analysis (GSEA), immune infiltration and mitochondrial metabolism, centered on these hub MitoDEGs. We also used real-time PCR and Spearman method to evaluate the relationship between plasma circulating cell-free mitochondrial DNA (ccf-mtDNA) levels and disease severity in AD patients.

**Results:**

MitoDEGs in AD were significantly enriched in pathways involved in mitochondrial respiration, mitochondrial metabolism, and mitochondrial membrane transport. Four hub genes (BAX, IDH3A, MRPS6, and GPT2) were selected to take part in the creation of a novel mitochondrial-based risk model for AD prediction. The risk score demonstrated excellent diagnostic performance in both the training cohort (AUC = 1.000) and the validation cohort (AUC = 0.810). Four hub MitoDEGs were also clearly associated with the innate immune cells’ infiltration and the molecular modifications of mitochondrial hypermetabolism in AD. We further discovered that AD patients had considerably greater plasma ccf-mtDNA levels than controls (U = 92.0, p< 0.001). Besides, there was a significant relationship between the up-regulation of plasma mtDNA and the severity of AD symptoms.

**Conclusions:**

The study highlights BAX, IDH3A, MRPS6 and GPT2 as crucial MitoDEGs and demonstrates their efficiency in identifying AD. Moderate to severe AD is associated with increased markers of mitochondrial damage and cellular stress (ccf=mtDNA). Our study provides data support for the variation in mitochondria-related functional characteristics of AD patients.

## 1 Introduction

Atopic dermatitis (AD) is one of the most prevalent chronic inflammatory skin diseases with a high burden of disease ranking among non-fatal diseases worldwide and is strongly associated with increased risks for asthma, rhinitis, and food allergy (1, 2). Regretfully, the majority of therapies concentrate on relieving symptoms because of our limited understanding of AD pathogenesis, which makes it difficult for early prevention and control of the disease. Along with the progression of AD, the deficiency of antioxidant capacity and the accumulation of large amounts of oxidants together build the oxidative stress (OS) milieu in AD (3–5). Existing studies have shown that the components of the OS milieu are distinct contributors to the epithelial immune microenvironment (EIME) in AD, which may be attributed to the dynamic cross-talk between reactive oxygen species (ROS) and type 2 immune inflammation (3, 6, 7). However, the unequivocal OS pathophysiological mechanism describing the development of AD remains poorly understood.

Mitochondria are particularly rich in the most metabolically active organs such as skin and serve a key role in providing energy and maintaining somatic homeostasis via oxidative phosphorylation (OXPHOS) and the generation of natural by-products of OXPHOS (mitochondria ROS) (8). Mitochondrion is a main source of intracellular ROS (9). Although epidermal differentiation and pigmentation benefit from mitochondrial ROS, perturbations of mitochondrial homeostasis are frequently reported in skin aging and skin cancer (8, 10, 11). In terms of mechanisms, impaired mitochondrial energetics in skin cells would accelerate a surge in mitochondria ROS production and mitochondrial DNA (mtDNA) mutations, thereby triggering a vicious cycle of OS and mtDNA damage (8, 12). It could explain abnormally elevated levels of OS markers (such as 8-OHdG) and an mtDNA variant localized to the MT-ND6 gene in the skin tissue and blood samples of AD patients (3, 13).

It is proposed that mitochondria play an important role in the pathogenesis of AD. Abnormal levels of mitochondrial metabolism and mitochondrial respiration in pro-oxidative situations have been gradually identified as a potential distinguishing characteristic of inflammatory illnesses like AD (5, 14, 15). Topical application of MitoQ, a mitochondrial targeting antioxidant, has been shown to effectively ameliorate AD-like eczema in mice through anti-inflammatory and antioxidant effects (14). It is necessary to uncover novel critical mitochondria-related genes, in light of aberrant mitochondria activity in the course of AD, to better understand the potential mechanism of AD and provide new ideas for molecular diagnosis and therapy for these patients. Through the setup of a large sample database and the development of Biochip, bioinformatic techniques have allowed us to gain an understanding of the components that contribute to disease at the multi-omics level. However, to the best of our knowledge, disease-specific biomarkers that correlate with AD have not been found using bioinformatics analysis of mitochondria-related genes.

In this work, we applied a combination of protein-protein interaction (PPI) network analysis and machine learning techniques to locate hub mitochondria-related differentially expressed genes (MitoDEGs) in the AD skin transcriptome based on the relevant microarray data from the Gene Expression Omnibus (GEO) database. Preliminary investigations were also conducted into potential correlations between hub MitoDEGs and the EIME of AD as well as mitochondrial metabolic function. In addition, it is essential to look for mitochondria-related indicators at the transcriptome level of AD blood to evaluate mitochondrial dysfunction. Circulating cell-free mitochondrial DNA(ccf-mtDNA) refers to “free-floating” non-encapsulated double-stranded DNA fragments that emerge from any cell type in the body (16). Damaged mitochondria often release ccf-mtDNA into the bloodstream, where it functions as a damage-related molecular pattern (DAMP) in intercellular communication and the cellular innate immune inflammatory response (17). Due to its high detectability in human bodily fluids (blood, urine, saliva), ccf-mtDNA has been identified as a promising biomarker for the estimation of related inflammatory diseases. Psoriasis and lupus are two examples of inflammatory skin diseases for which ccf-mtDNA are being actively investigated (16, 18, 19). We therefore chose plasma ccf-mtDNA as a biomarker of systemic mitochondrial damage and investigated the connection between ccf-mtDNA levels and the risk of AD subpopulations to further substantiate the involvement of mitochondrial dysfunction in AD etiology.

## 2 Materials and methods

### 2.1 Recruitment of participants and sample collection

57 participants (AD, n=38; healthy control, HC, n=19)were recruited from the Dermatology Department of the Second Affiliated Hospital of Harbin Medical University, Harbin, China. The Chinese criteria (20, 21) was used to diagnose AD participants, and the Eczema Area and Severity Index (EASI) criteria was used to assess the degree of clinical symptoms. Healthy subjects were defined by a lack of history of visible signs of skin damage that is indicative of AD. All participants who had EASI scores less than 8 points or who currently had a medical condition—such as an autoimmune or metabolic disease, malignant tumor, or hematological disease—were excluded. AD patients had not been treated with oral glucocorticoids or other immunosuppressive agents at least 1 month before study evaluation and blood draw. The demographic and clinical information of participants were summarized in **Table 1**. The study was authorized by the local ethics council (Medical Ethics Committee of the Second Affiliated Hospital of Harbin Medical University) and conformed with the Declaration of Helsinki principles. Following the signing of a written informed consent form by each participant, clinical data and samples were gathered for the study.

**Table 1:** Baseline demographics of AD and HC individuals.

	HC(n=19)	AD(n=38)	Statistical test	p value
**Female sex, n (%)**	11 (57.9%)	16 (42.1%)	X = 1.267	0.26
**Age (mean (SD))**	28.68 (2.76)	26.97 (3.198)	U = 270.5	0.125
**Ccf-mtDNA** **(log, (mean (SEM))**	3.42 (0.19)	4.61 (0.10)	t = 6.264	< 0.001
**EASI score (mean (SEM))**	–	21.96 (1.39)	t = 10.049	< 0.001
**IgE (mean (SEM))**	–	526.14 (71.45)	t = 5.964	< 0.001
**EO% (mean (SEM))**	–	5.62% (0.86%)	t = 0.723	0.474

AD, atopic dermatitis; HC, healthy controls; EO, eosinophils.

Before being sampled, the recruited individuals were told not to use body wash or lotion for 24 hours, nor to use topical treatments for 7 days. In addition, all participants had non-fasting peripheral blood drawn via venipuncture into EDTA tubes, and the blood was processed in four hours. Following extraction using the FICOLL separation method (Haoyang Biological Manufacture, Tianjin, China), supernatants were separated, aliquoted, and kept at -80°C until laboratory examination. The skin lesions, of 6 AD patients and 6 HCs were fixed with formaldehyde for subsequent laboratory examination. As previously mentioned, their peripheral blood mononuclear cells and supernatants were also acquired.

### 2.2 Dataset collection and preparation

Three AD and one psoriasis gene expression profiles, including RNA sequencing dataset GSE121212, microarray dataset GSE120721, GSE16161, and GSE109248, were taken from the NCBI GEO online public database (http://www.ncbi.nlm.nih.gov/geo/) (22). **Supplementary Table S1** provides a summary of the comprehensive baseline data. GSE121212, consisting of 27 AD lesional samples, 27 AD non-lesional samples, and 38 healthy samples, served as the training cohort, while GSE120721, which included 15 AD lesional samples and 22 healthy samples, GSE16161, including 9 AD lesional samples and 9 healthy samples, GSE109248, including 17 psoriasis samples and 14 healthy samples, were employed for external validation. To standardize these datasets, the R v4.3.0 “DESeq2” and “Limma” packages were utilized. All single data that lacked transcript IDs were eliminated. We selected the transcript ID with the average expression when a gene has several transcript IDs. Using the “Batch correction” method, GSE120721 and GSE16161 were combined into an expression matrix after the elimination of batch effects and normalization. For all ensuing downstream analyses, the raw gene expression data were quantile normalized and log2 transformed.

### 2.3 Identification of MitoDEGs and functional enrichment analysis

Using the R program “DESeq2,” DEGs between AD and HC in GSE121212 were assessed. Statistical significance was determined by |log2FoldChange| > 0.5 and an adjusted p-value< 0.05. To retrieve human genes with high certainty of mitochondrial localization, the mitochondrial protein database MitoCarta3.0 (http://www.broadinstitute.org/mitocarta) (23) was used. By overlapping DEGs and the genes localized in the mitochondria, MitoDEGs in AD were discovered. After that, up- and down-regulated MitoDEGs underwent pathway enrichment analyses using the R packages “clusterProfiler” and “org.Hs.eg.db” for Gene Ontology (GO) and Kyoto Encyclopedia of Genes and Genomes (KEGG), respectively, with a p-value< 0.05 being statistically significant.

### 2.4 PPI network analysis

The MitoDEGs were prepared for PPI analysis using the STRING database (https://string-db.org/) (24), and Cytoscape 3.9.1 was used to show the resulting interactions as a network. With Cytoscape 3.9.1, the “MCODE” plug-in was used to further identify the PPI network’s essential subnetwork. The primary screening parameters were max. depth = 100, K-core = 2, and node score cutoff = 0.2. Furthermore, using the “cytoHubba” plug-in of Cytoscape 3.9.1, each node gene was scored by 12 algorithms: EcCentricity, Closeness, Radiality, Betweenness, Stress, Clustering Coefficient, Edge Percolated Component (EPC), BottleNeck, Maximal Clique Centrality (MCC), Density of Maximum Neighborhood Component (DMNC), Maximum Neighborhood Component (MNC), and Degree. The key clusters identified from the MCODE plugin and the junction genes from the 12 algorithms of the cytoHubba plugin were merged for the following analysis.

### 2.5 Identification and validation of hub MitoDEGs

The training set GSE121212’s machine learning-based creation process for mitochondria-relate diagnostic markers was taken from an earlier article (25) and was described as follows: (1) To screen for critical variables that could differentiate AD from control situations, the random forest (RF) algorithm (26) was used, with a filter requirement of Mean Decrease Gini (MDG) greater than 0.25. (2) The merging genes found in the PPI network and these RF-screened significant genes intersected, and the resulting genes were chosen as candidate modeling genes. (3) Using the R “glmnet” packages, the least absolute shrinkage and selection operator (LASSO) logistic regression (27) was used to further reduce the range of potential modeling genes. Ultimately, the hub MitoDEGs in AD were chosen using ten-fold cross-validation to determine the optimum λ and the risk score for each sample was computed using the method that follows:


$$\text{risk score }=\;\sum_{i=1}^{\text{N}}\text{β}i\times Ei$$

where N, β, and E represent the total number of selected marker genes, the coefficient index of each gene calculated by LASSO regression, and the gene expression value of each gene, respectively.

Using the R package “rms,” a nomogram model (28) based on the differentially expressed hub MitoDEGs was built to calculate the diagnosis probability of AD patients. The receiver operating characteristic (ROC) curve analysis was used to show the prediction performance of hub MitoDEGs and risk score. The GSE109248 psoriasis dataset and the combined AD dataset (GSE120721 and GSE16161) were utilized as external validation cohorts to assess the model’s resilience and diagnostic capacity.

### 2.6 Gene set enrichment analysis

The important biological pathway changes of hub MitoDEGs in AD were found using GSEA (http://www.broadinstitute.org/gsea) (29). The predefined gene sets were chosen from the KEGG gene set (c2.cp.kegg.v2023.1.Hs.symbols.gmt) by the Java application from the Molecular Signatures Database (MSigDB) (http://software.broadinstitute.org/gsea/msigdb/). Maximum and minimum gene set sizes of 500 and 15 genes, respectively, were used to filter gene sets. Gene sets with p-value< 0.05 were deemed significantly enriched following 100 permutations.

### 2.7 Correlations between hub MitoDEGs and mitochondrial metabolism in AD

The genes involved in the mitochondrial metabolism were extracted and classified from MitoCarta3.0 database, and the correlations between mitochondrial metabolism and hub MitoDEGs were computed with the Mantel test and the Pearson correlation coefficient in AD non-lesional and AD lesional groups via the R package “ggcor”.

### 2.8 Immune infiltration analysis

Using RNA-seq or microarray data, the CIBERSORT algorithm, a deconvolution approach founded on the linear support vector regression principle, can determine the infiltration abundance of 22 immune cell types in a sample (30). Using the R package’s “CIBERSORT” algorithm, the abundance of 22 immune cell types in AD and healthy samples (GSE121212) was determined. Using Spearman’s rank correlation, the relationship between each hub MitoDEG and the 22 immune cells was examined and the results were displayed as lollipop charts and heatmaps.

The “GSVA” R package, which is widely used in immune infiltration-related bioinformatics research, employs the ssGSEA algorithm to evaluate the relative infiltration abundance of 28 immune cells in AD and normal skin tissues (31). The “ComplexHeatmap” package in R was used to create heatmaps and clustering analyses that illustrate the correlation.

### 2.9 Immunohistochemical verification

Extracted fresh skin tissues were fixed with 4% formaldehyde buffer overnight and 5-μm-thick sections were obtained from paraffinized specimens. Tissue sections were incubated at 60°C for 2 h before the dewaxing process. For antigen retrieval, the sections were autoclaved in a citric acid buffer (pH 6.0) at 115°C for 2 min and quenched in 0.3% H_2_O_2_ for 15 min for endogenous peroxidase activity. Then, sections were treated with immunol staining blocking buffer (Beyotime, Shanghai, China) for 30 min, and incubated overnight at 4°C with primary antibodies against IDH3A(Proteintech, Wuhan, China, dilution 1:200), BAX(Proteintech, Wuhan, China, dilution 1:2000), MRPS6(Bioss, Beijing, China, dilution 1:300), and GPT2(Proteintech, Wuhan, China, dilution 1:300). These sections were treated with HRP-conjugated secondary antibodies (ZSGB-BIO, Beijing, China) for 30 minutes at 37°C and the DAB substrate. Micrographs of the stained sections were captured by light microscopy (Zeiss Imager A2, Germany), and three fields were randomly selected for each skin tissue section. We then used the Image J v1.54f software (NIH, Wayne Rasband, USA) to measure the integrated optical density (IOD) value and the positive area of each visual field image. The average OD (AOD) of the positive sites (IOD/area) was used to evaluate the relative expression of the target genes.

### 2.10 DNA isolation and measurement of ccf-mtDNA content in plasma

Thawed plasma at room temperature was followed by centrifugation at 10000×g for 10 min to remove cells and cellular debris. According to the protocol of Serum/Plasma Free DNA Extraction Kit (Tiangen, Beijing, China), we isolated total DNA in plasma with 30 µl of elution buffer and quantified using spectrophotometric analysis at 260/280 nm in NanoPhotometer ^®^ P-Class (Implen, Westlake Village, CA, US). It is essential to have all the different samples under study adjusted to the same concentration (8-12ng/μL).

Quantitative analysis of the ccf-mtDNA content in human plasma by real-time PCR has been reported (32). Briefly, we first amplified MT-ND1 (GGCTATATACAACTACGCAAAGGC, GGTAGATGTGGCGGGTTTTAGG) to obtain purified PCR products from a control individual and calculated the copy number per 1μL of the purified DNA by the following equation (32):


$$\begin{array}{c} \frac{\text{Avogadro's constant}}{330\text{Da}\times 2\times \text{the size of the PCR fragment }\left(\;117\text{bp}\right)}\\ \times \text{Concentration of DNA}(\text{ng}/\text{μL}) \end{array}$$

The ccf-mtDNA copy number was reported as copies per microliter (copies/μL). We performed serial dilution of the purified PCR product by calculating copy numbers to create the standard curve. The reaction efficiencies of the standard curves ranged from 90% to 110%, with an ideal R^2^≈1. The crossing-point values from the testing samples were compared with the standard curve to quantify the DNA concentration.

The PCR reaction was performed using SYBR (SEVEN, Beijing, China) in a 7900HT Fast Real-Time PCR apparatus (Applied Biosystems, USA). Each reaction contained 10ng of template, 0.4μL of each primer(10μM), 5μL SYBR MIX, and 3.2μL of nuclease-free water. PCR program: initial denaturation at 95°C for 30 s, followed by 40 cycles consisting of 95°C in 15 s (melting) and 60°C for 15s (annealing and extension). The program ended with a melting curve analysis measuring fluorescence continuously from 60 to 95°C.

### 2.11 Statistical analysis

R (version 4.3.0) and IBM^®^ SPSS^®^ Statistics (version 19.0, Chicago, IL, USA) were applied for statistical analysis. The Shapiro-Wilk test (n ≤ 50) or the Kolmogorov-Smirnov test (n>50) was used to check the normality of the data. Categorical variables were compared using the chi-square test. The Mann-Whitney U test or Student’s t-test was used to test for differences between AD and HC for continuous variables with non-normal or normal distribution, respectively. Correlations between two variables were evaluated using Spearman’s rho or Pearson test. Statistical significance was set at p<0.05.

## 3 Results

### 3.1 Identification of MitoDEGs and functional enrichment analysis associated with AD

**Figure 1:**
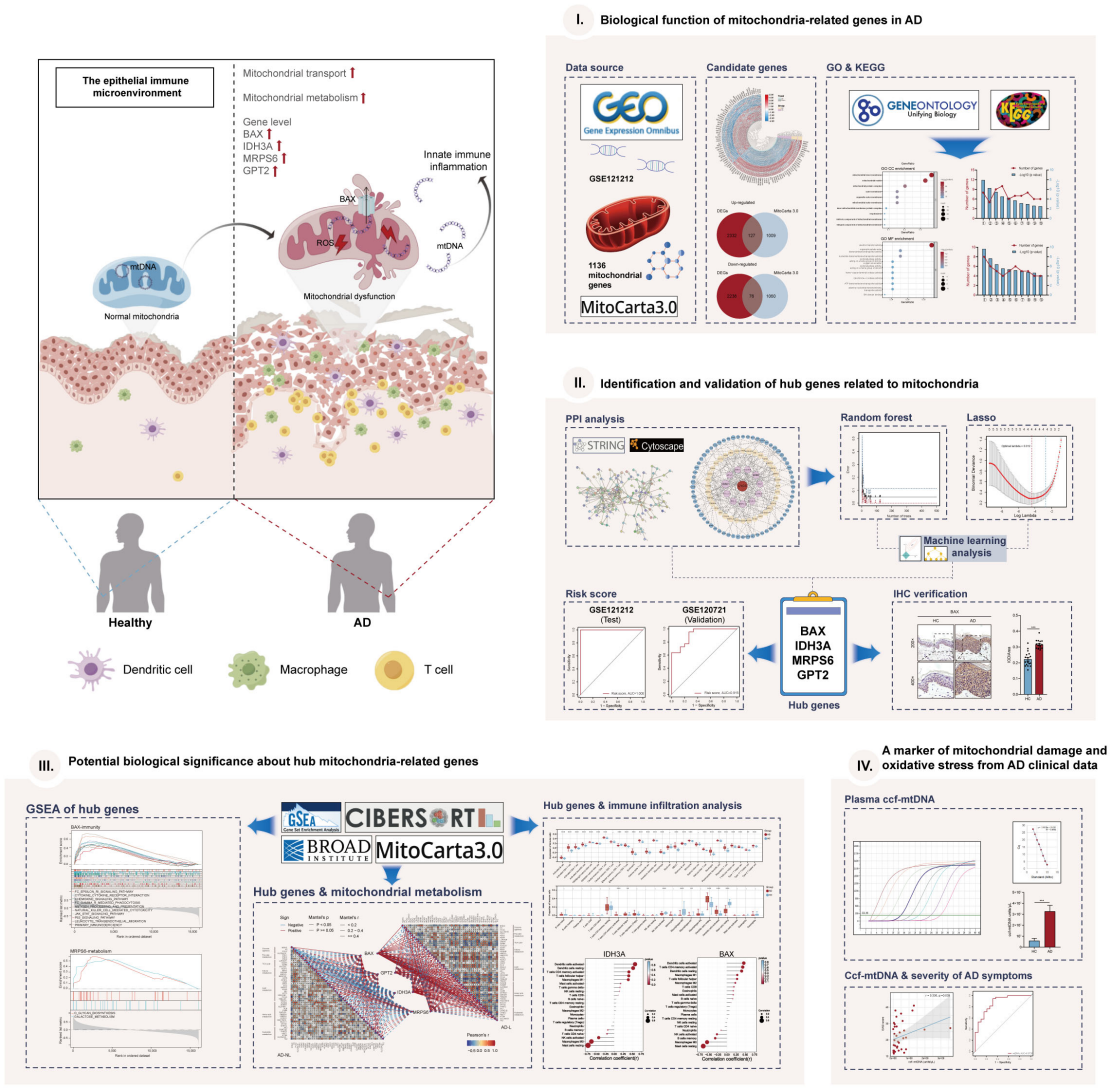
The graphical abstract and workflow of this study. By Figdraw.

**Figure 2:**
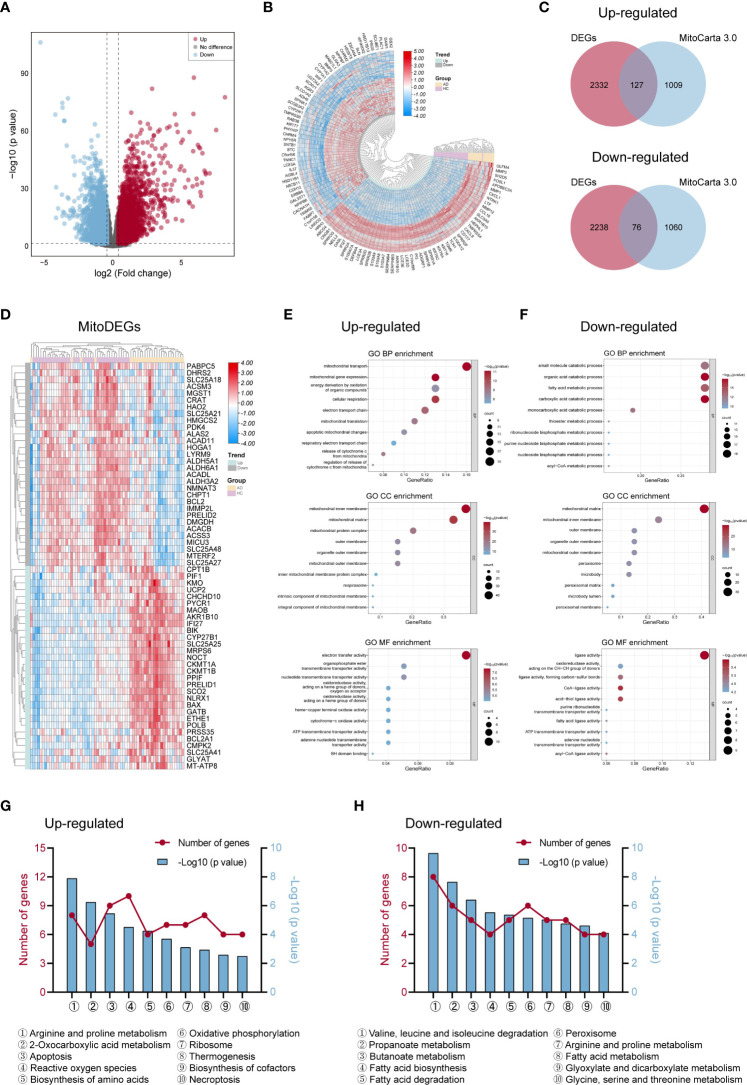
Differentially expressed genes linked to mitochondria and their roles in AD. **(A, B)** The volcano map **(A)** and heat map **(B)** of DEGs in AD and control groups. **(C)** Venn diagrams displayed the number of MitoDEGs chosen from MitoCarta 3.0’s mitochondria-related genes and up- and down-regulated DEGs. **(D)** The heat map of the expression of the top 60 MitoDEGs. **(E, F)** GO enrichment analysis of up-regulated **(E)** and down-regulated **(F)** MitoDEGs. **(G, H)** KEGG pathway analysis of up-regulated **(G)** and down-regulated **(H)** MitoDEGs. AD, atopic dermatitis; DEG, differentially expressed gene; MitoDEGs, mitochondria-related differentially expressed genes; GO, gene ontology; KEGG, kyoto encyclopedia of genes and genomes.

The workflow of this study was shown in **Figure 1**. We performed DEGs analysis on the GEO dataset GSE121212 by DESeq2, and the results showed that a total of 4773 DEGs were differentially expressed between AD and HC samples on the criteria of |log2FoldChange| >0.5 and p <0.05. The volcano plot of DEGs between the two groups was shown in **Figure 2A**, in which, 2459 genes were up-regulated and 2314 genes were down-regulated in the AD group. The heatmap representing the most significant DEGs was shown in **Figure 2B**. Taking the intersection of these DEGs with 1136 mitochondria-related genes retrieved from the MitoCarta3.0 database, 203 overlapped MitoDEGs (127 up-regulated and 76 down-regulated) were detected in AD skin tissue (**Figures 2C, D**).

GO and KEGG pathway analyses were performed to explore these MitoDEGs’ biological characteristics in more detail. **Figures 2E, F** displayed the MitoDEGs’ most enriched GO keywords, which include biological process, molecular function, and cellular component. The redox reaction, energy metabolism, mitochondrial respiration, and mitochondrial membrane transport were all linked to the up-regulated MitoDEGs for AD. The pathways related to metabolism, thermogenesis, apoptosis and necroptosis, OXPHOS, ribosome, peroxisome, and other processes dominated the most enriched KEGG pathways of the MitoDEGs (**Figures 2G, H**).

### 3.2 Identification of hub MitoDEGs from PPI analysis and machine learning

**Figure 3:**
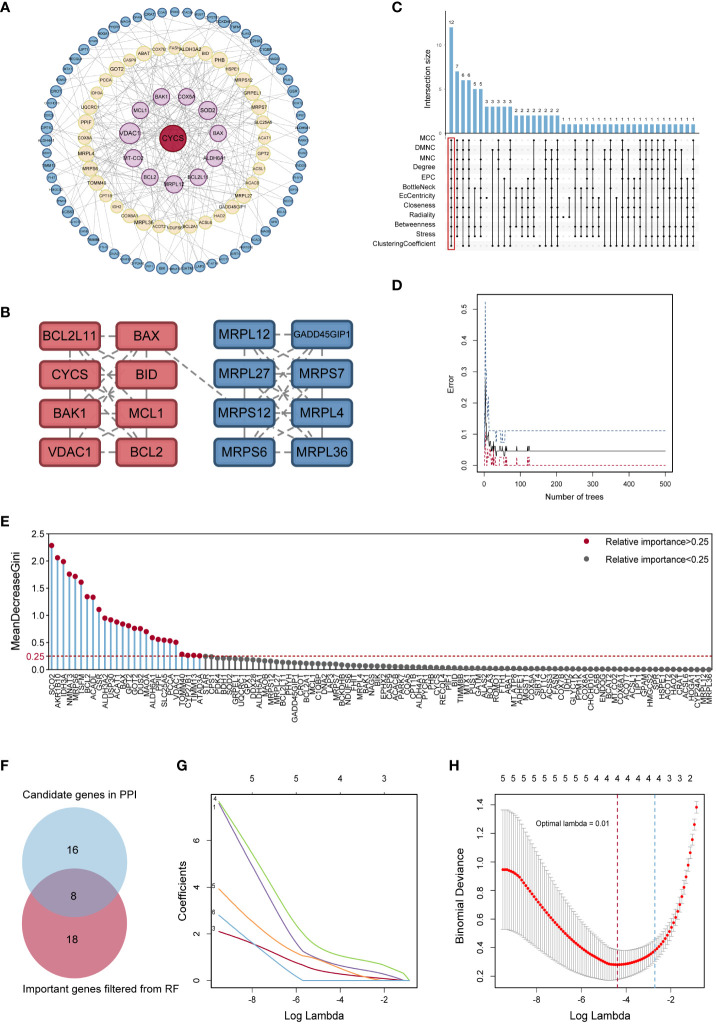
Identification of potential hub genes. **(A)** The PPI network of MitoDEGs. **(B)** MCODE highlighted a significant cluster of 16 genes. **(C)** The 12 node genes were intersected by the 12 algorithms of the cytoHubba plugin. **(D)** The model error and the number of RF trees in a correlation plot. **(E)** Relative significance of 26 MitoDEGs determined using RF. **(F)** Venn diagram representing the points where key gene variables from RF pre-screening and PPI analysis overlap. **(G)** Candidate hub MitoDEGs were screened out using LASSO regression. **(H)** Ten-fold cross-validation for tuning parameter selection in the LASSO regression. PPI, protein-protein interaction; RF, random forest; LASSO, least absolute shrinkage and selection operator.

The PPI network of MitoDEGs was analyzed using the STRING database and visualized as a network with the Cytoscape (**Figure 3A**). The MCODE plug-in of Cytoscape software was utilized to extract significant modules (gene clusters) from the PPI network, resulting in 16 candidate genes (**Figure 3B**). Meanwhile, 12 candidate genes were segregated from the PPI network using 12 algorithms of plug-in CytoHubba (**Figure 3C**). A total of 24 genes were obtained after the combination. In addition, based on the above 203 MitoDEGs, 26 genes were further selected as key variables capable of distinguishing AD and HC samples through RF algorithm analysis (MDG ≥ 0.25, p<0.05, **Figures 3D, E**). We eventually acquired 8 candidate hub genes for the final LASSO regression modeling to further narrow the gene number by intersecting the significant genes acquired via RF with the candidate genes discovered in the PPI network (**Figure 3F**). The results showed the lambda values ranged from 0.01027782 to 0.05484964. Plots for LASSO regression coefficients over different values and tenfold cross-validation for the penalty term were shown in **Figures 3G, H**. The lambda.min was confirmed as 0.01027782 where the optimal lambda resulted in 4 non-zero coefficients. In the end, 4 hub MitoDEGs identified by LASSO regression included BAX, IDH3A, GPT2, and MRPS6 (**Table 2**).

**Table 2:** The information of 4 hub MitoDEGs.

Gene symbol	Gene ID	Full name	Location	Function of the encoded protein
**IDH3A**	3419	NAD (+) 3 catalytic subunit alpha	Mitochondria	NAD(+)-dependent isocitrate dehydrogenases are thought to play a major role in the allosterically regulated rate-limiting step of the tricarboxylic acid cycle.
**BAX**	581	BCL2 associated X	Cytoplasm	BAX is reported to interact with, and increase the opening of, the mitochondrial voltage-dependent anion channel (VDAC), which leads to the loss in membrane potential and the release of cytochrome c and mtDNA.
**MRPS6**	64968	Mitochondrial ribosomal protein S6	Mitochondria	MRPS6 encodes a 28S subunit protein that belongs to the ribosomal protein S6P family. As a protein coding gene, MRPS6 has been implicated in numerous biological functions including mitochondrial translation and metabolism of proteins.
**GPT2**	84706	Glutamic-pyruvic transaminase 2	Mitochondria	This gene encodes a mitochondrial alanine transaminase, a pyridoxal enzyme that catalyzes the reversible transamination between alanine and 2-oxoglutarate to generate pyruvate and glutamate.

### 3.3 Efficiency of hub MitoDEGs screened by LASSO model

**Figure 4:**
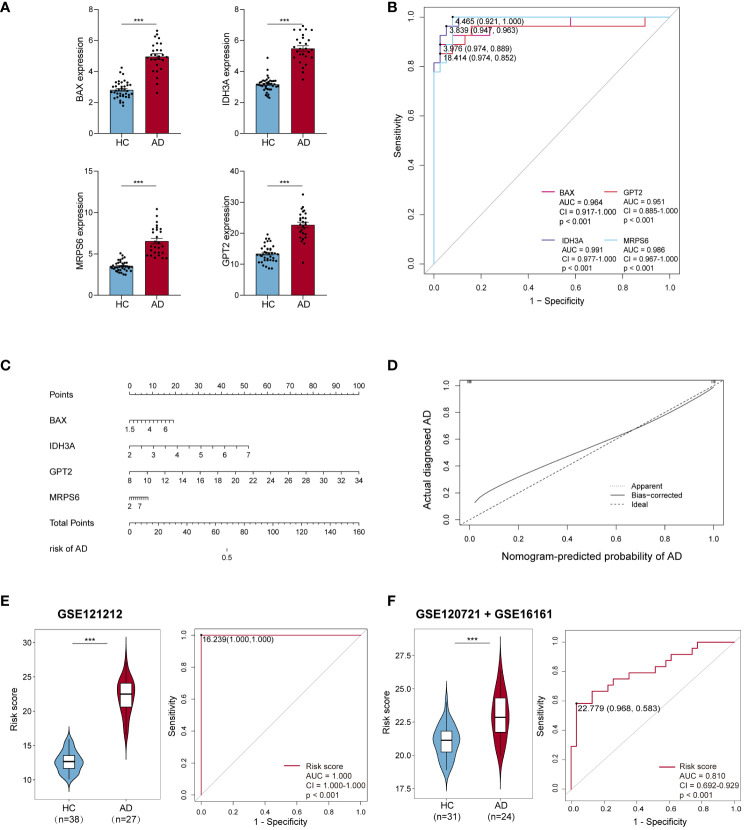
Efficiency of hub MitoDEGs. **(A)** Expression of the 4 hub MitoDEGs between AD and HC groups. **(B)** The ROC curve of four hub MitoDEGs for AD diagnosis. **(C)** The nomogram of LASSO regression. **(D)** The calibration curve of the hub genes model. **(E, F)** Distribution of risk scores constructed by four hub MitoDEGs between ADs and controls and ROC analysis of risk scores in GSE121212 **(E)** and GSE120721+GSE16161 **(F)**. Mean ± SEM, ***p<0.001. HC, healthy control; ROC, receiver operating characteristic.


The expression levels of these four hub MitoDEGs were considerably higher in both AD lesion and non-lesion samples compared to HC samples (**Figure 4A**; **Supplementary Figure S1A**). The algorithm below was used to calculate each patient’s risk score based on the expression of the four model genes:

Risk score = 1.246*IDH3A+0.793*BAX+0.746*MRPS6+0.363*GPT2

We noticed that the group with a high MitoDEGs score was more likely to develop AD than the group with a low score (**Figure 4E**; **Supplementary Figure S1C**). ROC curves with AUC values were constructed to evaluate the predictive power of diagnostic risk models for AD. As shown in **Figures 4B, E**, the AUCs of IDH3A, BAX, MRPS6, GPT2, and risk score for AD diagnosis were, respectively, 0.991, 0.964, 0.986, 0.951, and 1.000, indicating that the efficiency of these 4 hub MitoDEGs and the model were good. Additionally, a nomogram model was created to assess the diagnostic probability of AD using four hub MitoDEGs (**Figure 4C**). The best prediction performance was nearly matched by the nomogram-predicted AD, as demonstrated by the calibration curve (C-index=1) (**Figure 4D**). Interestingly, the diagnostic efficacy of these marker genes and the composition model chosen by LASSO regression remained good when the AD group was substituted with non-lesional skin samples (**Supplementary Figures S1B-D**). To increase the reliability of the result, the marker genes were further validated using the combined AD dataset. The expression and AUC values of the 4 hub MitoDEGs were shown in **Supplementary Figures S2A, B**. Similar to the findings in GSE121212, the risk score was still higher in the AD group, and the AUC of the probability value was 0.810 (**Figure 4F**). The nomogram calibration curves (C-index=0.909) also displayed obvious concordance between the predicted results and observations in GSE121212 (**Supplementary Figure S2C**). Additionally, these critical genes were tested using LASSO regression in the validation set for psoriasis to investigate the hub gene expression and the specificity of its model in AD. The distinction was that, in terms of both gene expression and ROC curve, the only element in the psoriasis validation set that matched the trend of the AD dataset was BAX (**Supplementary Figures S3A, B**). It didn’t seem that the risk score produced using the aforementioned formula was appropriate for psoriasis diagnosis (**Supplementary Figure S3C**).

### 3.4 Biological significance underlying hub MitoDEGs

**Figure 5:**
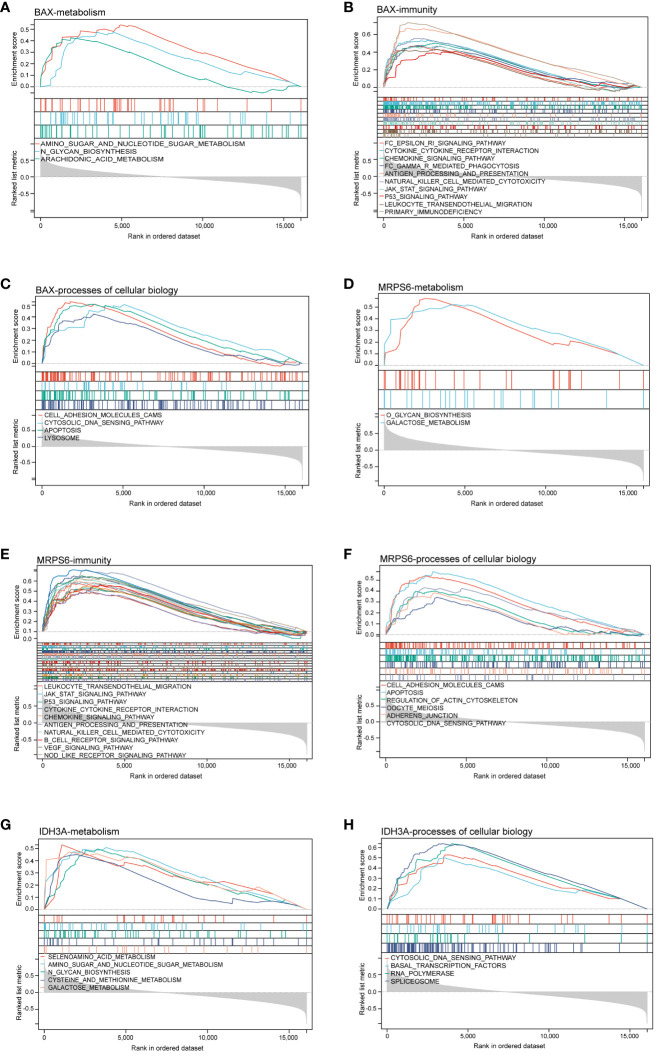
GSEA analysis of hub MitoDEGs. **(A–C)** BAX. **(D–F)** MRPS6. **(G, H)** IDH3A. GSEA, gene set enrichment analysis.

We applied the GSEA method to obtain a deeper insight into the function of hub MitoDEGs. GSEA showed that overexpression of BAX and MRPS6 were mainly involved in pathways related to glycometabolism and immunity, including N/O glycan biosynthesis, amino sugar and nucleotide sugar metabolism, arachidonic acid metabolism, galactose metabolism, NK cell-mediated cytotoxicity, cytokine-cytokine receptor interaction, chemokine, primary immunodeficiency, antigen processing and presentation, and the JAK-STAT signaling pathway (**Figures 5A, B, D, E**). The metabolic pathways linked to galactose metabolism, amino acid and amino sugar metabolism, and N glycan biosynthesis were similarly significantly enriched in the pathways that IDH3A changed (**Figure 5G**). Furthermore, they displayed numerous cellular biology processes related to cell adhesion, apoptosis, and the cytosolic DNA sensing pathway (**Figures 5C, F, H**). It’s noteworthy to note that we discovered a substantial correlation between the signaling pathways related to metabolism and immunity and the three mitochondria-related genes mentioned above.

**Figure 6:**
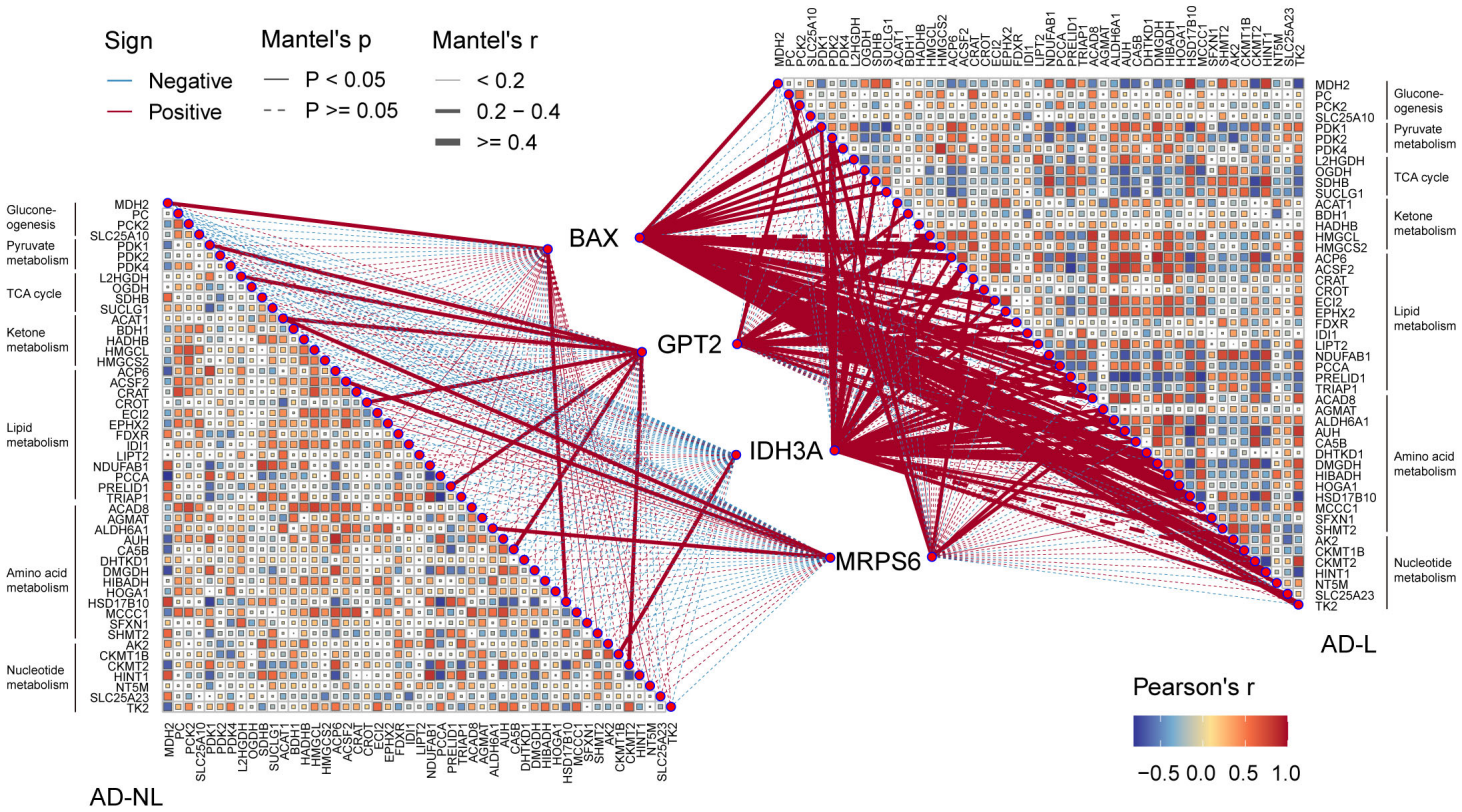
Correlation between hub MitoDEGs and mitochondrial metabolism. Color represents Pearson’s correlation coefficient r of each hub gene versus mitochondrial metabolism-related genes, with red color indicating a positive correlation (Pearson’s r< 0), and blue color indicating a negative correlation (Pearson’s r > 0). Statistical analysis was done with the Mantel test, with a full line indicating p value<0.05 and, a dotted line indicating p ≥0.05.

Thus, we investigated the possible relationship between mitochondrial metabolism and the four hub MitoDEGs. In the AD-lesional group, the hub genes developed a strong positive correlation with the majority of mitochondrial metabolic pathways, including pyruvate/ketone/lipid/amino acid/nucleotide metabolism, TCA cycle, and gluconeogenesis, as **Figure 6** illustrates. These findings suggested that the lesional skin tissue of AD may be undergoing biological alterations related to mitochondrial hypermetabolism, which these hub MitoDEGs may partially reflect.

### 3.5 Immune cell infiltration and the association between hub MitoDEGs and differential immune cells in AD

**Figure 7:**
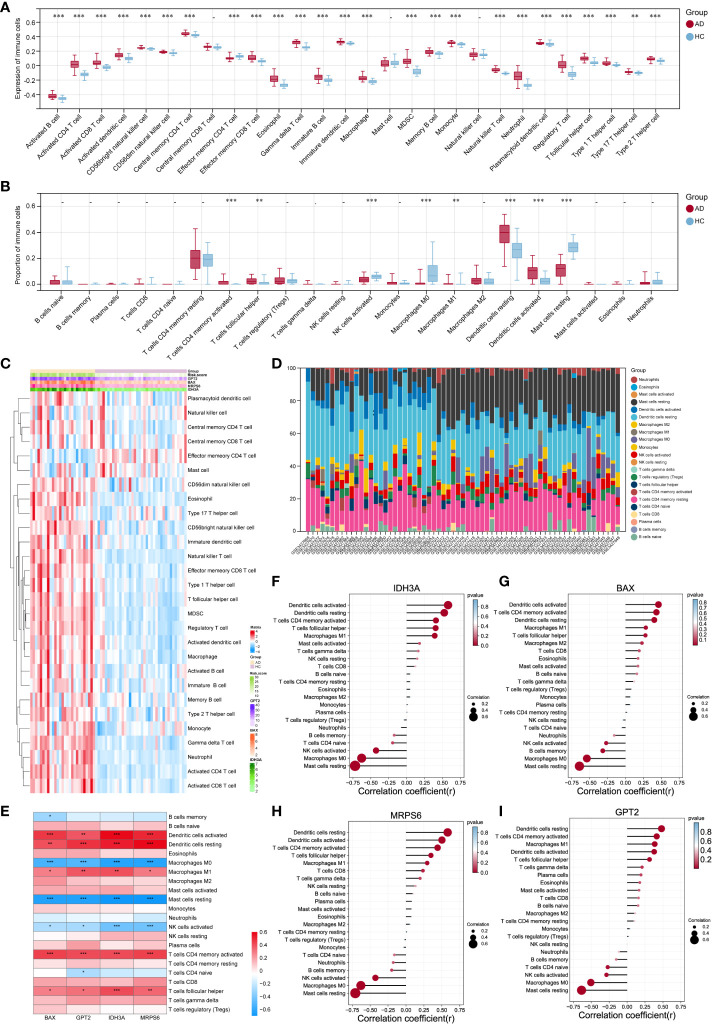
Examination of the infiltration of immune cells and the connection between hub genes and distinct immune cells in AD patients. **(A, B)** The violin plots showed the expression **(A)** of immune cells and their percentage **(B)** in the AD and HC tissues. **(C)** Heatmap of the expression of 28 immune cell types. **(D)** Stacked bar chart of the immune cell. **(E-I)** The link between immune cells and hub MitoDEGs in AD was depicted using the heat map **(E)** and lollipop plots, including IDH3A **(F)**, BAX **(G)**, MRPS6 **(H)** and GPT2 **(I)**. Mean ± SEM, *p< 0.05, **p<0.01, ***p<0.001.

SsGSEA and CIBERSORT were performed to understand the differences in immunological function better. The majority of immune cell subtypes were shown to be significantly expressed in AD skin tissue by ssGSEA analysis, indicating an environment of excessive immunological activation (**Figures 7A, C**). Moreover, CIBERSORT analysis revealed significant differences (p< 0.05) in the infiltration of eight immune cell types into skin tissue between the AD and HC groups. In particular, the AD group had significantly more activated memory CD4^+^ T cells, T follicular helper cells (Tfh), M1 macrophages, and resting/active dendritic cells (DC); in contrast, the HC group had significantly more activated NK cells, M0 macrophages, and resting mast cells (**Figures 7B, D**). We also further explored the correlation between these four hub MitoDEGs and immune cells (**Figure 7E**). **Figures 7F–I** demonstrates that hub MitoDEGs had a negative correlation with resting mast cells, activated NK cells, and M0 macrophages, but a positive correlation with innate and adaptive immune cells like DC, M1 macrophages, activated memory CD4^+^ T cells, and Tfh cells. These results suggested that the hub MitoDEGs could reflect immune cell infiltration in the skin tissue of AD patients.

### 3.6 Expression level of the hub MitoDEGs in skin tissue and peripheral blood of AD patients

**Figure 8:**
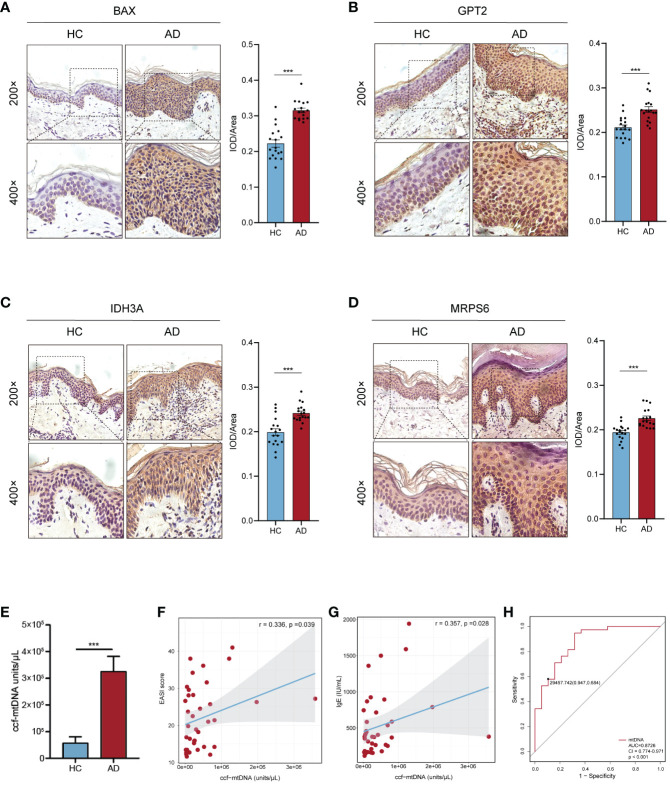
Confirmation of hub MitoDEGs expression and the diagnostic value of ccf-mtDNA in moderate to severe AD. **(A-D)** Expression of BAX **(A)**, GPT2 **(B)**, IDH3A **(C)**, and MRPS6 **(D)** in AD tissue and HC tissue. **(E)** Measurement of plasma ccf-mtDNA copy number levels between AD and HC groups. **(F, G)** Correlations between EASI score **(F)**, IgE **(G)**, and plasma ccf-mtDNA in AD patients. **(H)** The ROC curve of plasma ccf-mtDNA for AD diagnosis. Mean ± SEM, ***p<0.001. ccf-mtDNA, circulating cell-free mitochondrial DNA.

The AOD values of BAX, GPT2, IDH3A, and MRPS6 in AD skin tissue were considerably higher than those in HC skin tissue (p< 0.001), according to the results of skin tissue IHC between six AD patients and six healthy controls (**Figures 8A–D**). These outcomes agreed with the GSE121212 dataset’s findings in human skin tissues. These four hub genes were mainly located in the cytoplasm and nucleus of epidermal basal cells in HC skin tissues, where they had a light yellow or brown-yellow color. Nevertheless, the positive degree of gene expression progressively diminished or even became negative as one moved from the deep spinous layer to the stratum corneum. These four hub genes were unevenly expressed, with brownish-yellow or brown coloring across the cytoplasm of the entire epidermal layer in the lesions of AD patients. The four hub MitoDEGs with distinct differential AOD values were also analyzed for their associations with EASI scores in the AD group(**Supplementary Figure S4**). We did not find a significant correlation between EASI scores and BAX (rho = 0.321, p = 0.536), GPT2 (rho = -0.072, p = 0.892), IDH3A(rho = 0.147, p = 0.780) and MRPS6 (rho = -0.073, p = 0.891). Peripheral expression of 4 hubgenes was also validated in 12 recruiters mentioned above. Their expressions in the AD group tended to rise in comparison to the HC group (**Supplementary Figure S5A**). For the diagnosis of AD, the risk score obtained from the skin transcriptome and the blood transcriptome demonstrated similar accuracy and stability. The AUC of the probability value and the c-index were both 1.000 (**Supplementary Figures S5B-D**).

### 3.7 Correlation between plasma ccf-mtDNA and the severity of AD individuals

We evaluated the plasma ccf-mtDNA concentration in individuals who had moderate-to-severe AD. The findings showed that the peripheral plasma of people with moderate-to-severe AD had higher levels of ccf-mtDNA (455,533 ± 108,599 copies/μL) than did healthy people (57,705 ± 22,844 copies/μL) (U = 92.0, p<0.001) (**Figure 8E**). We next examined the relationship between the participants’ baseline characteristics and ccf-mtDNA in plasma samples (**Table 3**, **Figures 8F, G**). The concentration of ccf-mtDNA was found to be positively correlated with EASI scores (rho = 0.336, p = 0.039) and the levels of IgE (rho = 0.357, p = 0.028) when we included only participants with AD. We did not find a significant correlation between ccf-mtDNA concentration and age (rho = -0.232, p = 0.082), sex (rho = -0.161, p = 0.231), EO% (rho = 0.185, p = 0.267) (**Table 3**). We also did a ROC analysis to evaluate the diagnostic properties of ccf-mtDNA to identify AD cases (**Figure 8H**). The optimum cut-off point was greater than 29458, with a sensitivity of 94.7% and specificity of 68.4%. The area under the curve was 0.873 ± 0.05, p<0.001. An analysis was conducted on the relationship between the AOD values of the four hubgenes in the corresponding IHC results of six AD patients and the plasma ccf-mtDNA expression level. In AD patients, there was no link with other genes, but a significant positive association was found between the AOD value of BAX and the ccf-mtDNA copy number (rho = 0.934, p = 0.006) (**Supplementary Figure S6**).

**Table 3:** Correlation between ccf-mtDNA and clinical diagnostic indicators in AD individuals.

Parameters	rho	p value
Ccf-mtDNA		
**Age**	-0.232	0.082
**Sex**	-0.161	0.231
**EASI score**	0.336	0.039
**IgE**	0.357	0.028
**EO%**	0.185	0.267

AD, atopic dermatitis; Ccf-mtDNA, circulating cell-free mtDNA; EO, eosinophils.

## 4 Discussion

Although mitochondria can regulate many other cellular processes in skin cells such as energy metabolism, redox balance, growth/differentiation, and apoptosis, few studies have evaluated the role of mitochondria-related genes in the pathogenesis of AD (8). For the first time, we have comprehensively shown in this work the various pathobiological modifications of mitochondria in the AD environment, including gene expression levels, cellular infiltration, and biological pathways. We also screened out four mitochondria-related biomarkers in AD and checked their validity with machine-learning classifiers. Besides, the increased plasma ccf-mtDNA levels in AD patients indicated its role in the progress of AD. These findings may provide new insights into the AD pathogenesis.

Previous targeted microarray-based studies have demonstrated de-coordinated anti-oxidative response in AD epidermis (14). The fact that some down-regulated antioxidant genes support mitochondrial quality control in multiple ways is noteworthy. They regulate mitochondrial metabolism (mitophagy and oxygen homeostasis) through a series of cascade reactions (33–35), which in turn modulate mitochondrial stress response (reducing mitochondrial ROS clusters) (36), mitochondrial respiratory function (oxygen transport and cytochrome C oxidase maturation) (33, 37), and programmed cell death (ferroptosis) (38). Meanwhile, our results reveal that mitoDEGs participated in OHPHOS and redox reactions in addition to processes linked to mitochondrial membrane transport and small molecule catabolism, including amino acid metabolism, according to GO and KEGG analysis. These results extend our knowledge of mitochondrial activity in AD beyond its well-known role in bioenergetics.

Different from prior screening methods, the combined application of biological analysis and machine learning revealed IDH3A, BAX, MRPS6, and GPT2 as key MitoDEGs in AD. This was achieved in response to the growing scale and complexity of biological databases (39). The diagnostic utility of the potential biomarker genes and the risk model they comprised were also examined. The nomogram model and ROC curve results indicate that these four hub genes have favorable effects on AD diagnosis. In particular, the identification of these four mitochondria-related biomarkers from the blood transcriptome offers a promising avenue for the future development of non-invasive diagnostic methods. Remarkably, the risk model outperformed the individual four-hub MitoDEGs in terms of diagnosis accuracy. A diagnostic model constructed by multi-genes is more comprehensive and effective than a single gene.

We further deduced that there might be anomalies in mitochondrial biological processes in the epidermis of AD lesions, given that the altered protein expression patterns of these four hub genes are primarily seen in the superficial layer of the epidermis. BAX, a key apoptosis regulator that mediates the decisive step of mitochondrial outer membrane permeabilization, is recruited and co-assembled with BAK which is a pro-apoptotic member of the BCL2 family to induce apoptotic pore expansion, mtDNA release, and activation of cGAS/STING signaling pathway (40). According to reports, pro-inflammatory factor release via the STING signaling pathway is induced by tissue-released mtDNA, aggravating atopic processes from the skin to the gut (41). Interestingly, our results highlighted that increased BAX expression in AD promoted ccf-mtDNA accumulation in extracellular fluid (plasma), which may provide strong support for the mitochondria-related hypothesis that BAX mediates pathophysiological changes in AD EIME through a mtDNA-induced proinflammatory mechanism. Taking part in the oxidative decarboxylation of isocitrate into α-ketoglutarate, IDH3A is an essential enzyme that produces ATP in the mitochondrial tricarboxylic acid (TCA) cycle (42). According to past research, AD patients’ no-lesion skin has a higher energy metabolism than healthy skin (43). This finding may be related to glutamine’s attempt to speed up the TCA cycle turnover rate by reversing the keratinocytes’ significantly lower levels of citrate/isocitrate expression in non-lesional areas of AD (15, 44). Without a doubt, OS and mitochondrial ROS overproduction are progressively triggered by the activation of mitochondrial energy metabolism (45). Our findings could support the idea that IDH3A is involved in the pathophysiology of AD by dysregulating mitochondrial activity and isocitrate metabolism. Research on MRPS6 and GPT2 has mostly examined their role in increasing tumor cell proliferation and metastasis (46–49), with little attention paid to their role in AD. Unexpectedly, we found that the up-regulation of hub MitoDEGs was not statistically significantly correlated with the severity of AD individuals. Perhaps these initially explored mitochondrial-related biomarkers do not seem to help assess the severity of AD. Additional research is required, given the insufficient validation sample size.

Mitochondria not only integrate cellular metabolism and physiology, but they are also a major source of immunity (50). Moreover, immune cell metabolism and the activation of associated signaling pathways depend on mitochondria. For example, amino acid metabolism is a key modulator of redox balance in immune cells and supports essential metabolic reprogramming for immune cell activation (51). A crucial TCA cycle enzyme called fumarate causes the cytosol to release mtDNA, which in turn changes the mitochondrial network to activate innate immunity (52). GSEA analysis showed BAX, IDH3A, and MRPS6 were importantly involved in pathways related to metabolism, immunity, and cellular biology. After more research, we discovered that four hub MitoDEGs are strongly associated with the mitochondrial metabolic pathways in AD, which include pyruvate/ketone/lipid/amino acid metabolism, TCA cycle, and gluconeogenesis. All of the findings showed that the pathophysiology of AD and mitochondrial immunity and metabolism interact and overlap. Recently, Thomas et al. reported the single-cell transcriptomics and proteomics results obtained through skin aspiration blisters and highlighted the prominent role of DC and macrophages in maintaining the typical immune microenvironment of AD (53). This is following our findings when examining AD immune cell infiltration using the CIBERSORT or ssGSEA methods. Due to a study on lesional DC in AD patients, DC polarity expands or activates memory T cells, which in turn maintains the state of inflammation, rather than directly driving differential T-cell subset responses (54). Uncertainty surrounds the involvement of MI/M2 macrophages in AD; the majority of studies have shown an increase in both macrophage subtypes (55, 56). Furthermore, macrophage-produced CCL13 is regarded as a new inflammatory cytokine in the AD EIME (54). In our study, Tfh cells, a subset of CD4^+^ T cells, were enriched in AD patients over controls. A negative relationship between IL-10^+^ Breg cells and Tfh cell differentiation in children with extrinsic AD has been observed (57), which may promote researchers focusing on the function of Tfh cells. Interestingly, there was a close relationship between the above-mentioned immune cells and the hub MitoDEGs, which promoted our understanding of the interaction among mitochondria and innate immune cells in the EIME of AD patients.

That is why we monitor if mtDNA, the bridge connecting mitochondrial OS damage and innate immune inflammatory processes, makes a difference in AD patients. Wang et al. recently reported that children with AD have higher levels of ccf-mtDNA in their peripheral plasma when compared to HCs (41), yet there is a shortage of information on adult AD patients. Our results refine this section and re-emphasize the important role of ccf-mtDNA in AD pathogenesis. When OS-damaged mitochondria are released into the cytoplasm, interstitial space, and circulation, mtDNA can function as a damage-associated molecular pattern that disrupts mitochondrial autophagy (58) and programmed cell death (59), in addition to inciting a cascade of uncontrollable inflammatory reactions (17). These studies provided more context for the rising amount of mt-DNA in adult AD patients’ peripheral plasma. Our findings, however, did not support the hypothesis that the patient’s age or the level of mt-DNA were related. This finding may have been caused by the limited sample size and the absence of a vertical design.

A few other limitations need to be noted as well. Our study first built the risk model using a tiny sample of public databases, and the verification part was comparatively weak. An independent prospective cohort study with a large sample of clinical data is necessary. Furthermore, while we have detected differences in the expression of hub MitoDEGs and ccf-mtDNA in AD patients, the possible mechanisms of their interaction with the EIME remain unclear. Our next research will concentrate on the recruitment and activation of these four genes, particularly BAX, in the innate immune cells of the AD EIME, as well as their function in controlling mtDNA cytoplasmic escape.

## 5 Conclusion

To summarize, we established a novel mitochondrial-based molecular signature that takes into account IDH3A, BAX, MRPS6, and GPT2. Our study combined bioinformatics analysis and machine learning to increase our understanding of the crosstalk relationship among these key genes, AD immune infiltration and mitochondrial metabolic function. In addition, we found that plasma ccf-mtDNA may be a key indicator of AD progression, providing evidence of mitochondrial OS damage during the advancement of AD in adult patients with moderate-to-severe AD. Our results may provide a new research trajectory for AD pathogenesis.

## Data availability statement

The datasets presented in this study can be found in online repositories. The names of the repository/repositories and accession number(s) can be found in the article/**Supplementary Material**.

## Ethics statement

The studies involving humans were approved by Medical Ethics Committee of the Second Affiliated Hospital of Harbin Medical University. The studies were conducted in accordance with the local legislation and institutional requirements. Written informed consent for participation in this study was provided by the participants’ legal guardians/next of kin.

## Author contributions

HY: Writing – original draft, Conceptualization, Data curation, Formal analysis, Writing – review & editing. JL: Conceptualization, Data curation, Writing – original draft. JY: Project administration, Writing – review & editing. XS: Project administration, Writing – review & editing. CW: Formal analysis, Writing – original draft. BB: Funding acquisition, Project administration, Writing – review & editing.
