# Supplementary material for: Screening mitochondria-related biomarkers in skin and plasma of atopic dermatitis patients by bioinformatics analysis and machine learning
Source: Front Immunol. 2024 May 7;15:1367602. doi: 10.3389/fimmu.2024.1367602 (PMC11106410; doi:10.3389/fimmu.2024.1367602)
Supplement: Supplementary file 1 [file Table_1.docx]

Supplementary Material

| Supplementary Table S1. **Baseline information regarding the selected datasets** | | | | |  |
| --- | --- | --- | --- | --- | --- |
| **GEO datasets** | **GSE121212** | **GSE120721** | | **GSE16161** | **GSE109248** |
| **Disease** | Atopic dermatitis | Atopic dermatitis | | Atopic dermatitis | Psoriasis |
| **Platform** | GPL16791  Illumina HiSeq 2500 (Homo sapiens) | GPL570 [HG-U133_Plus_2] Affymetrix Human  Genome U133 Plus 2.0 Array | | GPL570 [HG-U133_Plus_2] Affymetrix Human  Genome U133 Plus 2.0 Array | GPL10558  Illumina HumanHT-12 V4.0 expression beadchip |
| **Sample type** | RNA-seq | Microarray | | Microarray | Microarray |
| **Sample species** | Homo sapiens | Homo sapiens | | Homo sapiens | Homo sapiens |
| **Sample source** | Skin tissue | Skin tissue | | Skin tissue | Skin tissue |
| **Disease status** | Untreated | Untreated | | Untreated | Untreated |
| **N** | 92 | 37 | | 18 | 31 |
| **Group** |  |  | |  |  |
| Non-lesional | 27 | - | | - | - |
| Nesional | 27 | 15 | | 9 | 17 |
| Normal | 38 | 22 | | 9 | 14 |
| **Attribute** | Training cohort | Validation cohort | | Validation cohort | Validation cohort |
|  | | |  | |  |

Figure legends

Figure S1. **Identification of hub MitoDEGs related to non-lesional AD using LASSO regression.**

1. Expression of the 4 hub MitoDEGs between AD-NL and HC groups. (B) The ROC curve of four hub MitoDEGs and risk score for AD-NL diagnosis. (C) Distribution of risk scores constructed by four hub MitoDEGs between AD-NLs and controls. (D) The calibration curve of the hub genes model. Mean±SEM, *p < 0.05, **p <0.01, ***p <0.001.

Figure S2. **Validation of the marker genes in AD validation cohort.**

1. Expression of the 4 hub MitoDEGs between AD and HC groups in GSE120721 and GSE16161. (B) The ROC curve of four hub MitoDEGs for AD diagnosis. (C) The calibration curve of the hub genes model. Mean±SEM, *p < 0.05, **p <0.01, ***p <0.001.

Figure S3. **Validation of the marker genes in psoriasis validation cohort.**

1. Expression of the 4 hub MitoDEGs between psoriasis and HC groups in GSE109248. (B) The ROC curve of hub MitoDEGs for psoriasis diagnosis. (C) Distribution of risk scores constructed by four hub MitoDEGs between psoriasis and controls. Mean±SEM, *p < 0.05, **p <0.01, ***p <0.001.

**Figure S4. Correlations between EASI score and IOD/Area of 4 hub MitoDEGs in 6 AD patients.**

**Figure S5. Validation of the marker genes in perpheral blood of AD patients.**

1. Expression of the 4 hub MitoDEGs in peripheral blood of AD and HC groups. (B) The ROC curve of peripheral blood hub MitoDEGs for AD diagnosis. (C) Distribution of risk scores constructed by four hub MitoDEGs in peripheral blood between ADs and controls. (D) The calibration curve of the peripheral blood hub genes model. Mean±SEM, *p < 0.05, **p <0.01, ***p <0.001.

**Figure S6. Correlations between plasma ccf-mtDNA expression level and IOD/Area of 4 hub MitoDEGs in 6 AD patients.**
