# Supplementary figures and images for: Screening mitochondria-related biomarkers in skin and plasma of atopic dermatitis patients by bioinformatics analysis and machine learning
Source: Front Immunol. 2024 May 7;15:1367602. doi: 10.3389/fimmu.2024.1367602 (PMC11106410; doi:10.3389/fimmu.2024.1367602)

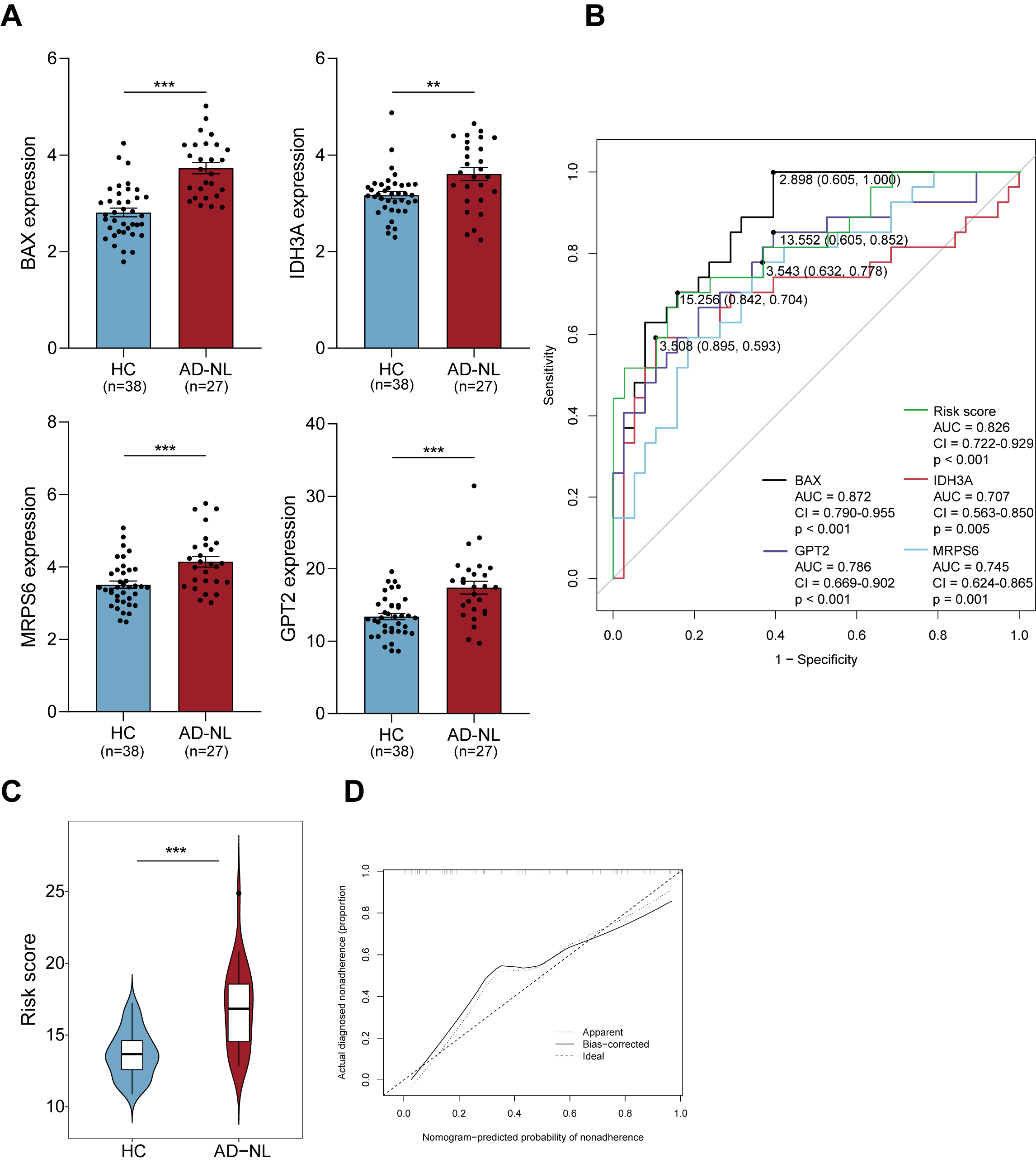

Supplement: Supplementary file 2 [file Image_1.tif]

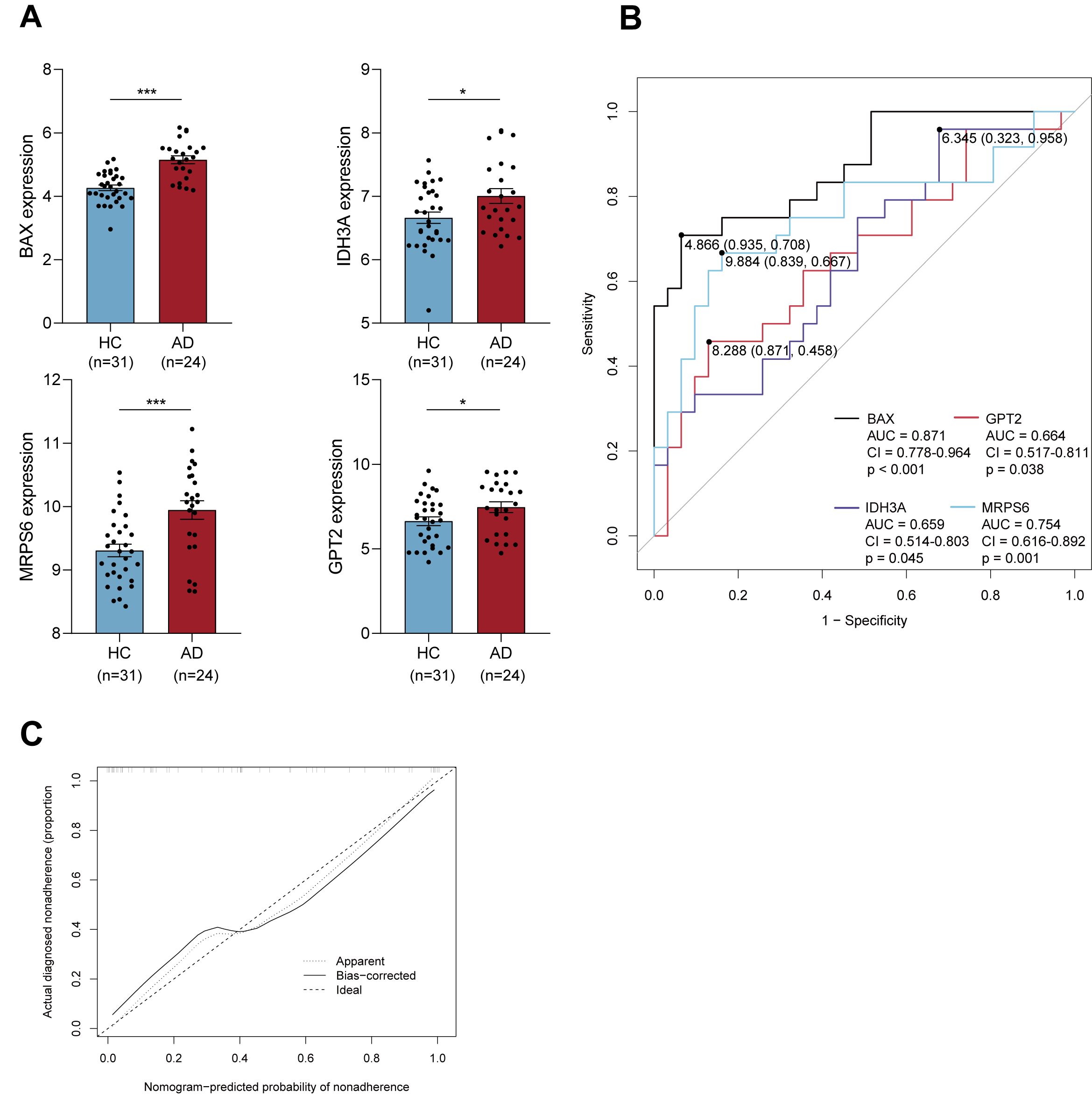

Supplement: Supplementary file 3 [file Image_2.tif]

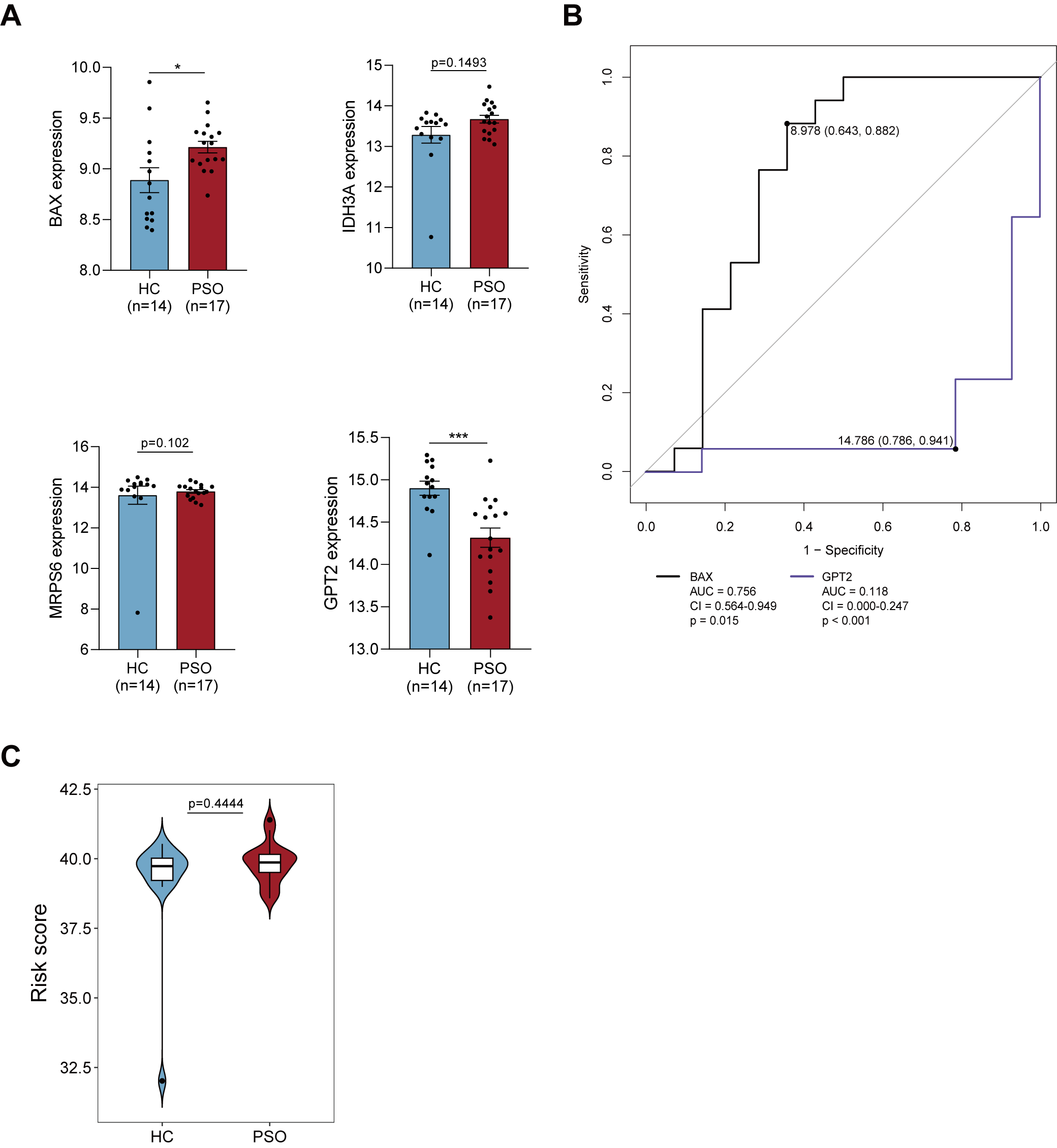

Supplement: Supplementary file 4 [file Image_3.tif]

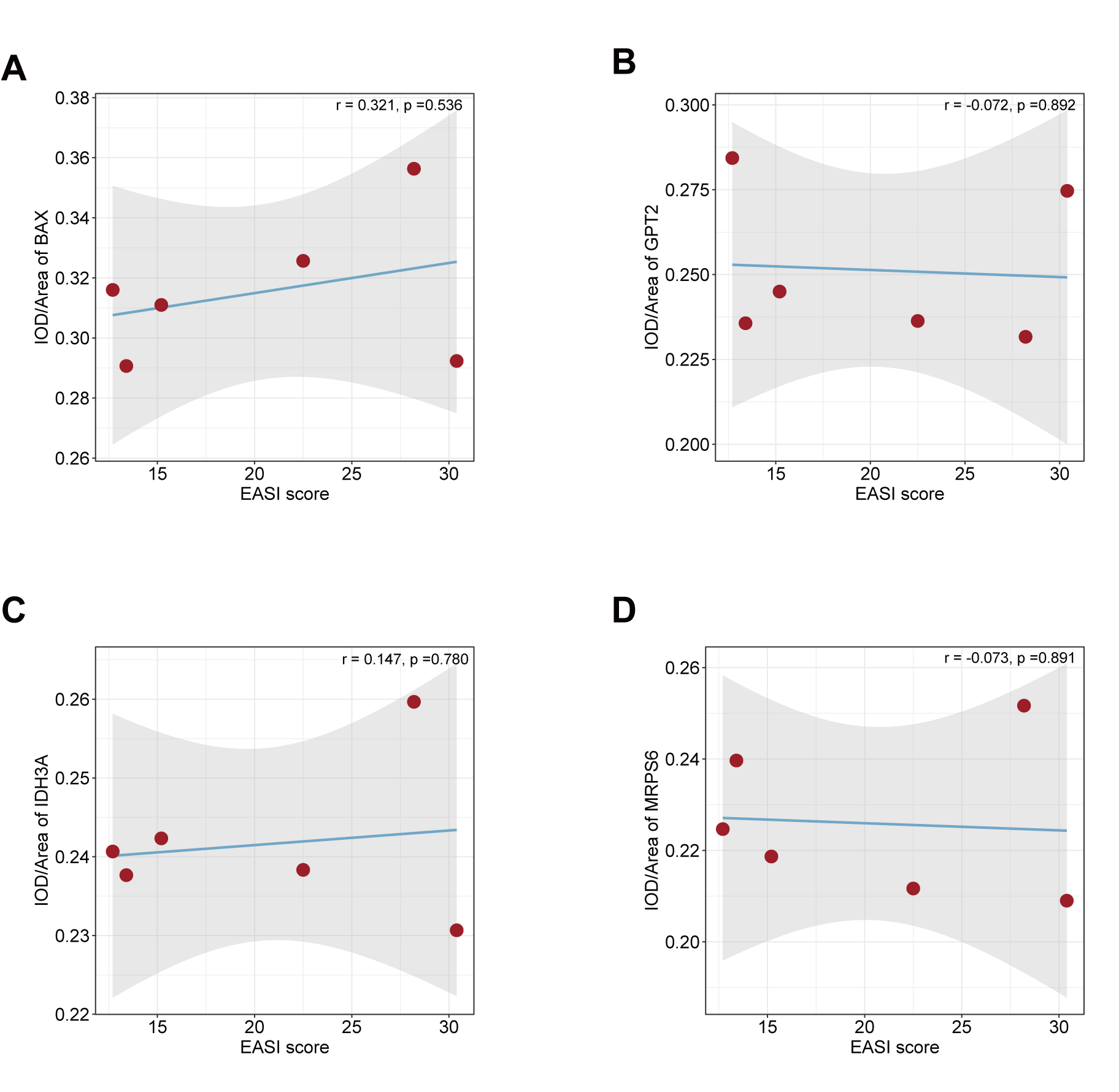

Supplement: Supplementary file 5 [file Image_4.tif]

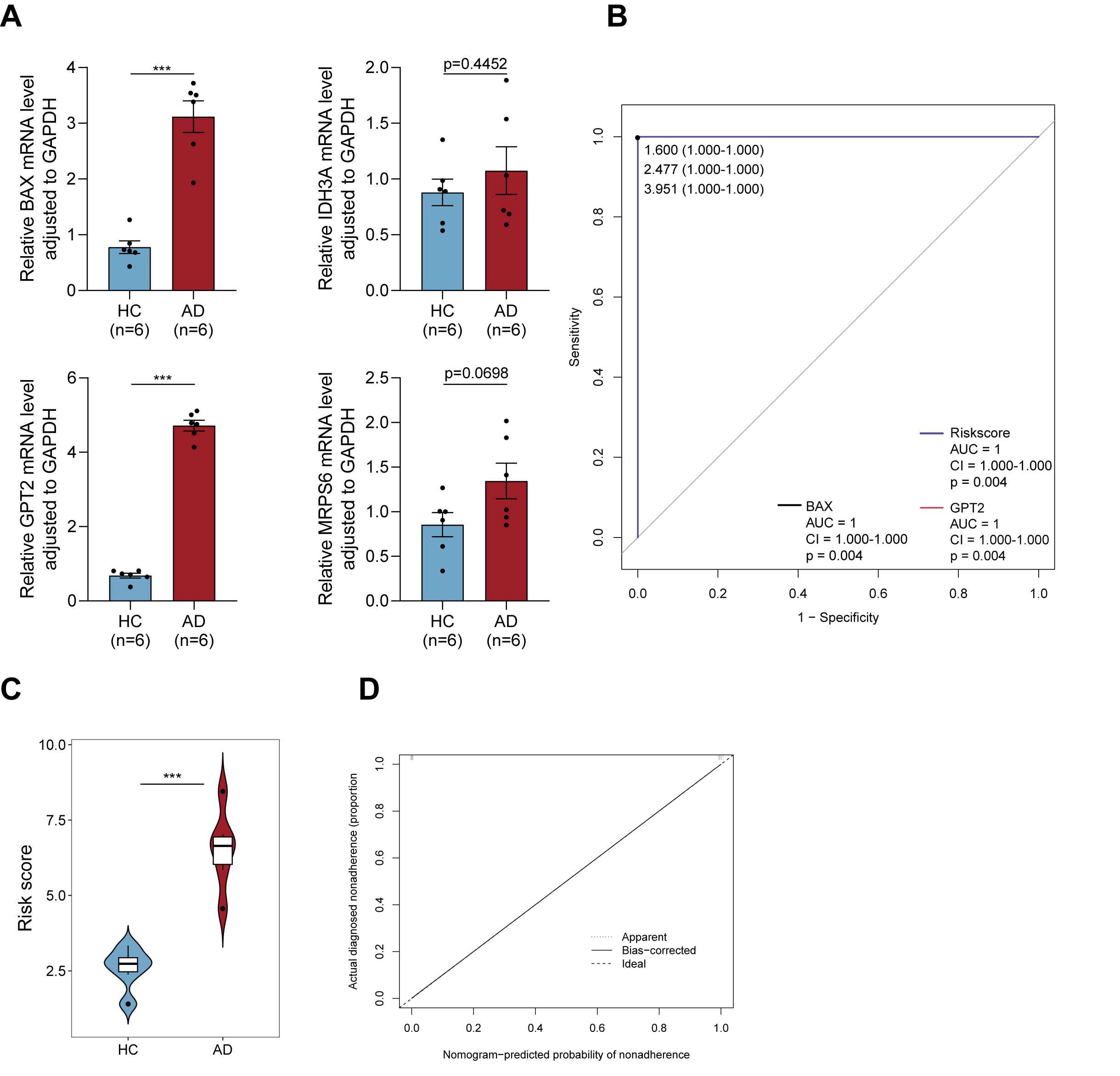

Supplement: Supplementary file 6 [file Image_5.tif]

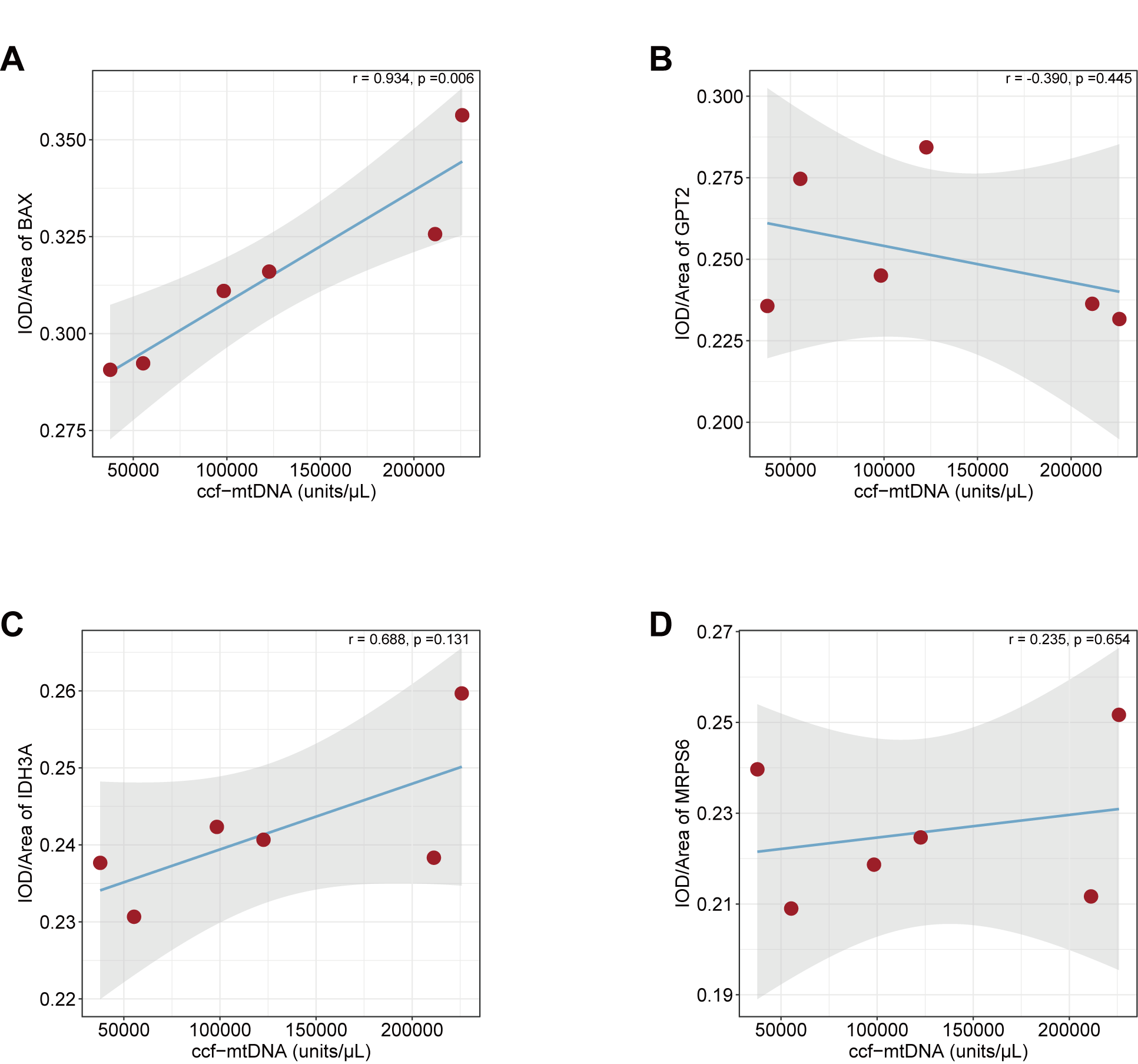

Supplement: Supplementary file 7 [file Image_6.tif]
